# Cost-effectiveness and budget impact of the microprocessor-controlled knee C-Leg in transfemoral amputees with and without diabetes mellitus

**DOI:** 10.1007/s10198-019-01138-y

**Published:** 2020-01-02

**Authors:** Alexander Kuhlmann, Henning Krüger, Susanne Seidinger, Andreas Hahn

**Affiliations:** 1 Center for Health Economics Research Hannover (CHERH), Leibniz Universität Hannover, Otto-Brenner-Straße 7, 30159 Hannover, Germany; 2 Otto Bock HealthCare Products GmbH, Vienna, Austria

**Keywords:** Knee prosthesis, Transferomal amputation, Microprocessor-controlled knee C-Leg, Cost-effectiveness, Economic evaluation, Markov model, I19

## Abstract

**Background:**

The safe use of a prosthesis in activities of daily living is key for transfemoral amputees. However, the number of falls varies significantly between different prosthetic device types. This study aims to compare medical and economic consequences of falls in transfemoral amputees who use the microprocessor-controlled knee joint C-Leg with patients who use non-microprocessor-controlled (mechanical) knee joints (NMPK). The main objectives of the analysis are to investigate the cost-effectiveness and budget impact of C-Legs in transfemoral amputees with diabetes mellitus (DM) and without DM in Germany.

**Methods:**

A decision-analytic model was developed that took into account the effects of prosthesis type on the risk of falling and fall-related medical events. Cost-effectiveness and budget impact analyses were performed separately for transfemoral amputees with and without DM. The study took the perspective of the statutory health insurance (SHI). Input parameters were derived from the published literature. Univariate and probabilistic sensitivity analyses (PSA) were performed to investigate the impact of changes in individual input parameter values on model outcomes and to explore parameter uncertainty.

**Results:**

C-Legs reduced the rate of fall-related hospitalizations from 134 to 20 per 1000 person years (PY) in amputees without DM and from 146 to 23 per 1000 PY in amputees with DM. In addition, the C-Leg prevented 15 or 14 fall-related death per 1000 PY. Over a time horizon of 25 years, the incremental cost-effectiveness ratio (ICER) was 16,123 Euro per quality-adjusted life years gained (QALY) for amputees without DM and 20,332 Euro per QALY gained for amputees with DM. For the period of 2020–2024, the model predicted an increase in SHI expenditures of 98 Mio Euro (53 Mio Euro in prosthesis users without DM and 45 Mio Euro in prosthesis users with DM) when all new prosthesis users received C-Legs instead of NMPKs and 50% of NMPK user whose prosthesis wore out switched to C-Legs. Results of the PSA showed moderate uncertainty and a probability of 97–99% that C-Legs are cost-effective at an ICER threshold of 40,000 Euro (≈ German GDP per capita in 2018) per QALY gained.

**Conclusion:**

Results of the study suggest that the C-Leg provides substantial additional health benefits compared with NMPKs and is likely to be cost-effective in transfemoral amputees with DM as well as in amputees without DM at an ICER threshold of 40,000 Euro per QALY gained.

**Electronic supplementary material:**

The online version of this article (10.1007/s10198-019-01138-y) contains supplementary material, which is available to authorized users.

## Introduction

Patients suffering from peripheral vascular disease (PVD) and/or diabetes may experience a transfemoral amputation—a major surgery—as a long-term consequence of their condition. Most of these patients require chronic medical interventions and medication anyway. As amputation is the treatment of last resort, the majority of patients have often endured re-vascularization attempts and/or long-standing in- and outpatient treatments of vascular and diabetic ulcers, or oftentimes even toe or forefoot amputations. Many patients also present other secondary conditions such as cardio-vascular and/or cerebro-vascular diseases. In Germany, the proportion of age-adjusted major amputations (above-ankle to hemipelvectomy) with diagnosed diabetes mellitus declined by 30.9% within 9 years (2005–2014), whereas minor amputations in this cohort increased by 25.4%. [1] Another publication reported comparable trends for the period 2005–2015: − 43% for hip joint/femoral amputation and − 36.9% for knee and lower leg amputation [2].

An amputation is a tremendous incision in personal life that affects the patient’s health and quality of life for the rest of his/her lifetime. Providing the most suitable individual prosthetic support for disability compensation and regaining self-sufficient participation in normal/social life are the primary rehabilitation goals after transfemoral amputations [3]. The safe use of a prosthesis in activities of daily living is key for transfemoral amputees [4]. Several clinical and biomechanical studies as well as two systematic reviews [4–15] investigated the safety of using the microprocessor-controlled knee joint C-Leg (C-Leg). Compared with non-microprocessor-controlled (mechanical) knee joints (NMPK), the C-Leg reduced the number and frequency of stumbles and falls of up to 80% [6, 8, 9]. In addition, four studies [16–19] that analyzed the health-related quality of life (HRQoL) in prosthesis users found improvements in the HRQoL of 14–38% when patients were using the C-Leg instead of NMPKs.

However, production costs of C-Legs are higher than those of conventional NMPKs and C-Legs require additional funding by the payer, when they are reimbursed. Therefore, it is important to investigate whether the price of the C-Leg is justified by the additional benefits. Four studies [16–18, 20] have assessed the cost-effectiveness of the C-Leg compared to NMPKs. Three studies were from Europe [16–18] and one study from the US [20]. Results varied from the C-Leg-dominating NMPKs in a patient cohort with an average age of 45–46 years old (societal perspective) [18] to an ICER of close to 90,000 Euros per quality-adjusted life year (QALY) gained in a cohort of amputees aged 40+ years old at the time of first prosthesis (payer’s perspective) [17]. Only one study included the medical and economic consequences of falling, which had a major impact on the cost-effectiveness of the C-Leg [20].

Due to the heterogeneous cost-effectiveness results and model assumptions in international economic studies and differences in cost structures, conclusions regarding the cost-effectiveness of C-Legs in Germany cannot be drawn from results of international studies. In addition, none of the economic studies investigated the impact of comorbidities on the cost-effectiveness of C-Legs. Several epidemiological studies found an improved survival in patients without diabetes mellitus (DM) compared to patients with DM over a longer time horizon [21–24]. Differences in all-cause mortality may have a major impact on the cost-effectiveness, since the benefits of the C-Leg add up over time. Therefore, the aim of our study is to assess the cost-effectiveness of the C-Leg compared to NMPKs in transfemoral amputees without DM and transfemoral amputees with DM from the perspective of the German SHI.

For German statutory health insurance companies, the information on the budget impact of new interventions is (currently) more important than estimates of the cost-effectiveness, since information on the budget impact is more relevant for short-to-midterm budget plans of the companies. So far, no German budget impact analysis of C-Legs does exist. Hence, another objective of this study is to predict the impact of C-Legs on the budget of the German SHI in 2020–2024.

## Methods

A decision-analytic model was developed in Microsoft EXCEL 2016 to perform a cost-effectiveness and budget impact analysis of C-Legs vs. NMPKs for transfemoral amputees with or without DM in Germany.

### Model structure

**Fig. 1:**
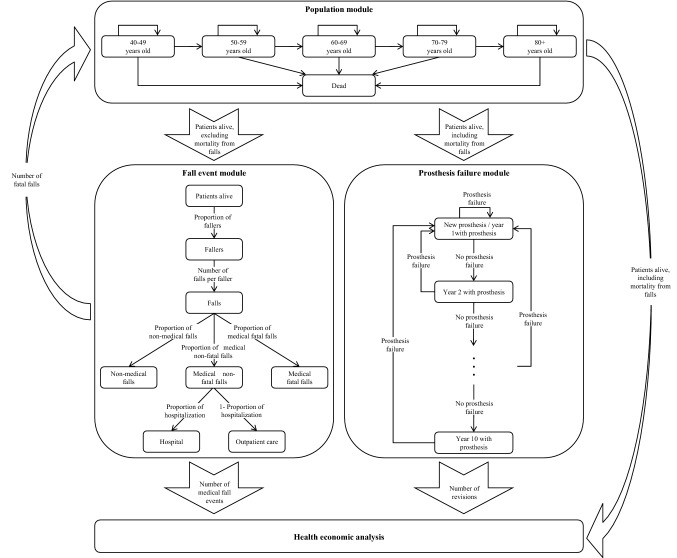
Structure of the decision-analytic model

The decision-analytic model consists of three structural modules: population, fall events, prosthesis failure. The population module simulates the survival and aging of recently transfemoral amputated patients who received their initial leg prosthesis. It includes five age groups: 40–49 years old, 50–59 years old, 60–69 years old, 70–79 years old and 80+ years old. The population is updated yearly (model cycle length = 1 year). Annual survival of amputees is computed in a multi-step process. First, prosthesis users who die from non-fall-related causes exit the simulation. The average annual population size is then calculated by summing up the number of survivors at the start and at the end of each year and dividing it by two (= half cycle correction). Based on the average annual population size, the fall events module—a simple decision tree—calculates the annual number of fall-related events, including fatal falls. In the fall events module, patients can either experience a fall or not. Falls may require medical attention or do not affect the health of the faller. Medical falls are further classified as fatal medical falls or non-fatal medical falls, which can lead to hospitalization or outpatient treatment (see Fig. 1). Finally, the population module corrects the number of surviving amputees (at the end of the year) for fatal falls and calculates the new average annual population.

The prosthesis failure module calculates the number of prosthesis revisions based on the estimated average annual population. The number of prosthesis revisions is a key input for determining the costs of prosthesis usage. It consists of a series of tunnel states (year 1 with prosthesis, year 2 with prosthesis, etc.) which track the number of years of surviving amputees since the initial prosthesis fitting or the last prosthesis revision to determine the time of the next prosthesis revision.

Figure 1 shows the structure and interactions of the different modules and the contribution of each module to the health economic analysis.

### Cost-effectiveness analysis

The cost-effectiveness analysis focuses on the projected number of new prosthesis users in 2020 who are 40+ years old. Cost-effectiveness was calculated separately for patients with or without DM. The time horizon of the analysis was 25 years. Clinical and economic benefits were evaluated from the perspective of the German SHI. Quality-adjusted life years (QALY) gained served as primary, prevented medical falls, hospitalizations and fatal falls as secondary outcomes. Costs and QALYs were discounted by 3% according to the recommendations of the Institute for Quality and Efficiency in Health Care (IQWiG) [25].

### Budget impact analysis

The budget impact was computed for the time period of 2020–2024 (time horizon of 5 years) from the perspective of the German SHI. Costs were not discounted [25]. The analysis included amputees aged 40+ years old who received the initial leg prosthesis between 2000 and 2024 and was performed separately for patients with and without DM. In total, 125 incident cohorts (five age groups; 25 years) entered the budget impact model. Surviving incident leg prosthesis users of the period 2000–2019 constituted the prevalent patient cohorts in the budget impact model. Methods for the projection of incident leg prosthesis users are described in the segment epidemiology.

In the base case analysis, we made the following assumptions regarding the market share of C-Legs:

- 2000–2019: all eligible transfemoral amputees receive NMPKs
- 2020–2024: 100% of incident prosthesis users receive C-Legs; 50% of NMPK prevalent users, whose leg prosthesis wears out, switch to C-Legs and the other half get a revised NMPK

Other market shares were applied in scenario analyses.

### Estimation of the number of prosthesis users

To date, there is no German leg prosthesis registry. Hence, we estimated the number of leg prosthesis users based on observed amputation incidence rates in 2005–2017. We performed a linear regression on the logged incidence rates to estimate time trends between 2005 and 2011 as well as 2012 and 2017. Incidence rates were then projected for the periods 2000–2004 and 2018–2024, respectively. Case numbers were calculated using demographic data. Finally, the annual number of new prosthetic users was determined as follows:

$${\text{IPU}}_{\text{noDM}} = {\text{TA}} \cdot (1 - {\text{pTA}}_{\text{DM}} ) \cdot (1 - {\text{mTA}}_{\text{noDM)}} ) \cdot {\text{pSF}} \cdot {\text{pSHI,}}$$

$${\text{IPU}}_{\text{DM}} = {\text{TA}} \cdot {\text{pTA}}_{\text{DM}} \cdot ( 1 {\text{ - mTA}}_{\text{DM}} ) \cdot {\text{pSF}} \cdot {\text{pSHI}} .$$

IPU_noDM_: incident prosthesis users without DM; IPU_DM_: incident prosthesis users with DM; TA: transfemoral amputations; pTA_DM_: proportion of transfemoral amputees with DM; mTA_noDM_: 30 day mortality in amputees without DM; mTA_DM_: 30 day mortality in amputees with DM; pSF: proportion of successful fitting; pSHI: proportion of Germans in the SHI.

Estimates of time trends and results of the projections are presented in the supplementary material.

### Data sources

Input parameter values were either obtained from public accessible databases, published peer reviewed articles or in case of prostheses prices and prosthesis survival times from Ottobock (manufacturer of the C-Leg and one of the world’s leading producers of NMPKs). A literature review was performed in PubMed, Cochrane Library and Google Scholar. References of identified studies were manually searched for additional publications. Prosthesis subject-specific publications in prosthetic and orthotic journals where identified manually. In addition, results of a literature review of a recently published health economic study [20] were considered. Input parameters are summarized in Table 1.

**Table 1:**
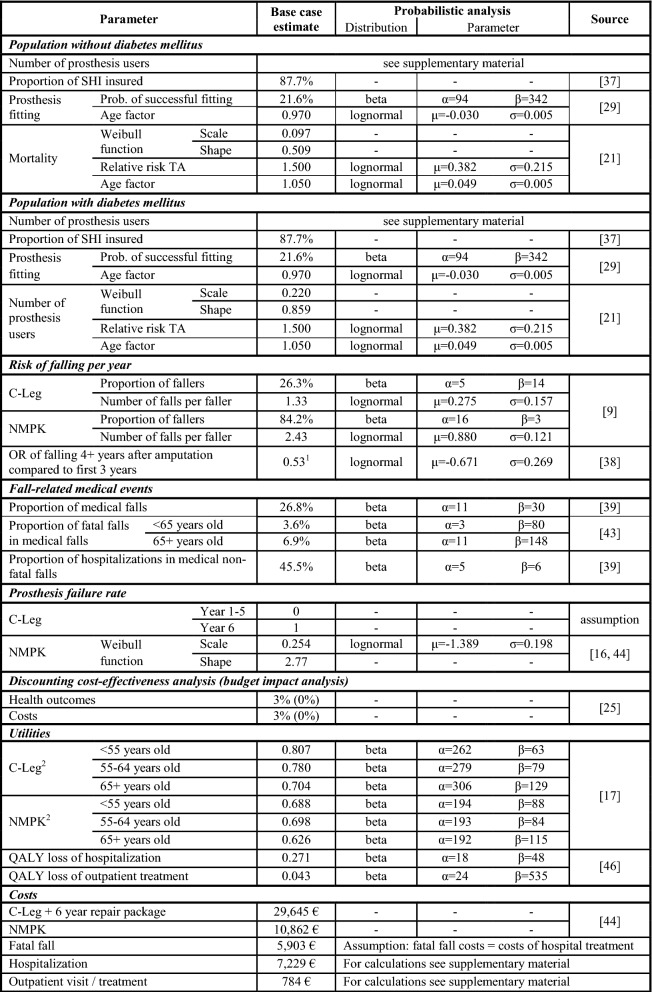
Model parameters

^1^An annual decline of $\frac{{1 - OR_{ < 4y\,vs\,4 + y} }}{{3 - OR_{ < 4y\,vs\,\,4 + y} }}$ in the proportion of fallers is assumed during the first 4 years after amputation. Base case values for the ORs of falling for 2, 3 and 4 + years compared with the first year after amputation are 0.81, 0.62, 0.43

^2^Utility in 50–59 years old: average of < 55 and 55–64 years old; utility in 60–69 years old: average of 55–64 and 65+ years old

#### Epidemiology

The annual number of transfemoral amputations were taken from official DRG statistics (OPS codes 5-864.3-5-864.7) [26]. Demographic data were obtained from the Federal Statistical Office of Germany [27, 28]. We identified one German study that reported midterm survival of lower limb amputees [21]. Other German studies only reported in-hospital-mortality [2]. Icks et al. [21] provided survival data for patients with and without DM up to 5 years after amputation. We fitted Weibull distributions to extrapolate data up to 10 years after amputation and assumed a fixed mortality rate from the tenth year onwards. 30 day mortality data which were used in the estimation of the annual number of leg prosthesis were also taken from the study of Icks et al. [21].

Several studies investigated prosthesis use and prosthesis fitting rates in lower limb amputees [29–36]. However, most studies [30–32, 34–36] had a small sample size and/or did not report outcomes for different amputation levels. Davie-Smith et al. [29] as well as Resnik et al. [33] found fit rates in transfemoral amputees of 21.6% and 19.2%, respectively. While Davie-Smith et al. [29] analyzed age effects separately for transfemoral and transtibial amputations, Resnik et al. [33] reported only a combined age coefficient. Hence, we applied data from Davie-Smith et al. [29]. Estimated age effects of both studies were comparable though (0.97 vs. 0.98) [29, 33].

In 2018, around 72.8 Mio Germans (87.7%) were insured in the SHI [37].

#### Risk of falling and fall-related medical events

In our model, the risk of falling depended on the prosthesis type, while the probability of a medical event following a fall was assumed to be independent of that type. We identified five clinical trials [4, 8–12] that compared the number of falls of MPK versus NMPK usage. Two of these trials [9, 12] provided patient level fall outcomes, while the other studies only reported average fall numbers over a specific time period. Due to the small sample size (*n* = 8) of the study by Wong et al. [12], we populated our model with data from Kahle et al. [9]. We excluded two outliers from the sample due to implausible model outcomes in 40–49 years old: for NMPKs, fall-related mortality would have been higher than the estimated all-cause mortality in 40–49 years old. Both outliers were younger than 40 years old and, therefore, did not belong to the target population of the analysis.

Long-term risk of falling in prosthesis users has not been evaluated yet. Based on data of lower limb amputees [38], we assumed that the proportion of fallers declined over time. According to Miller et al., the odds of experiencing a fall in the past 12 months decreased by 47% in patients who were amputated 4+ years ago [38]. We assumed an annual linear decline in the proportion of fallers (represented by $\frac{{1 - OR_{ < 4y\,vs\,4 + y} }}{{3 - OR_{ < 4y\,vs\,\,4 + y} }}$) in the first 4 years followed by a stable proportion.

Falls may either require medical attention or they do not affect the health of the faller (Fig. 1) [39]. We identified only one study that reported fall-related injuries in lower limb amputees [39]. According to Wong et al. [39], 26.8% of all falls had medical consequences. Chen et al. [20] calculated a lower proportion of medical falls (10.4%) based on three studies [40–42] in the elderly general US population. We tested this value in the sensitivity analysis. Hospitalization probability was 45.5% in Wong et al. [39] which was comparable to the figure of 40% applied by Chen et al. [20]. Fatal falls did not occur in the study by Wong et al. [39]. Possible reasons could be the small sample size or the design of the study (self reported outcomes). However, one subject died due to unknown causes and 12 subjects were lost to follow-up [39]. Hence, we applied the same fatal fall probabilities as Chen et al. [20]: 3.6% (of medical falls) in < 65 years old and 6.9% (of medical falls) in 65+ years old [43]. We tested different assumptions in the sensitivity analysis.

#### Prosthesis failure rates

The price of the C-Leg includes a 6-year maintenance package, which covers all repair costs. We assumed that C-Legs were replaced directly after the maintenance package had expired. For NMPKs, such a maintenance package does not exist. We calculated a mean NMPK survival time of 3.5 years based on data from Ottobock [44]. Brodtkorb et al. [16] found an increasing risk of NMPK failure over time and estimated a Weibull distribution. We also applied a Weibull distribution to determine yearly failure probabilities of NMPKs. The shape parameter of the distribution (2.77) was taken from Brothkorb et al. [16]. The scale parameter (0.2543) was calculated based on the shape parameter and the mean survival time of 3.5 years. Brodtkorb et al. [16] estimated a lower mean survival time of 2 years, which would result in a higher number of NMPK revisions. Nair et al. [45] analyzed prosthetic episodes in 100 transfemoral amputees. Over a time horizon of 10 years, transfemoral amputees needed 0.98 new prostheses and 2.31 major repairs. Based on this data, we calculated a mean prosthesis survival time of 7.11 years for new prostheses and a mean survival time of 2.26 years for new prostheses and major repairs combined. We tested the impact of both survival times on the outcomes in the sensitivity analyses.

#### Utilities

We identified four studies [16–19] which reported utilities for C-Leg and NMPK users. Three studies [16–18] used the EQ-5D 3L and one study [19] the SF-36/SF-6D. Brodtkorb et al. [16] derived utility values directly from the visual analog scale of the EQ-5D which is not the standard method for generating utilities and does not reflect preferences of the society. We obtained utility values from Cutti et al. [17], since this study had the largest ample size and was the only study that reported utilities for different age groups. Utility values were comparable to findings of Gerzeli et al. [18].

We found one study [46] which reported utility decrements for fall-related medical events. Hartholt et al. [46] collected HRQoL data at 2, 5 and 9 months after the fall incident for hospitalized and non-hospitalized patients. We used graph digitizing software (PlotDigitizer 2.6.3) to extract numerical data from plotted utility scores [46] of all patients presenting to the emergency department. Reported utility scores at 9 months after the fall incident were used to calculate utility decrements for hospitalized and non-hospitalized patients. Utility decrements were extrapolated up to 1 year. Long-term HRQoL reductions of fall events were not included in the evaluation.

#### Costs

The price year of the study was 2019. Average prices of C-Legs and NMPKs were calculated based on data from Ottobock [47]. The price of the C-Leg included a 6-year maintenance package. Costs of medical falls were estimated using a micro-costing approach. Resource utilization of hospitalized cases included hospital treatment, inpatient rehabilitation treatment and outpatient treatment after hospitalization. We assumed that costs of fatal falls were equal to the costs of hospital treatment. Costs of outpatient treatment were obtained from a German health economic study of osteoporosis patients [48] and adjusted for inflation based on the German consumer price index [49]. Long-term costs of falling were not included in the evaluation. A detailed list of cost components and cost calculations are provided in the supplementary material.

In the sensitivity analyses, we tested the impact of higher fall costs on the health economic outcomes. For this, we used results of US cost studies [50–52] adjusted for price level differences between the US and German healthcare sectors [53] and inflation [49].

### Sensitivity analyses

Univariate and a probabilistic sensitivity analyses (PSA) were performed to investigate the impact of changes in individual input parameter values on model outcomes and to explore parameter uncertainty. The PSA was run with 10,000 iterations. Base case estimates, probability distributions including parameters and references are summarized in Table 1.

## Results

### Falls and fall-related events

In amputees without DM, the predicted incidence rates of falls were 178 per 1000 person years (PY) among C-Leg users and 1102 per 1000 PYs among NMPK users. In amputees with DM, incidence rates of falls were 203 per 1000 PYs and 1201 per 1000 PYs, respectively. The major decrease in falls resulted in substantially reduced incidence rates of fall-related medical events among amputees using C-Legs compared with NMPK users: 3 vs. 17 fatal falls and 20 vs. 134 hospitalizations per 1000 PYs in amputees without DM as well as 3 vs. 18 fatal falls and 23 vs. 146 hospitalizations per 1000 PYs in amputees with DM. On average, C-Leg users without DM gained 1.96 life years (LYs) and C-Leg users with DM 0.55. Health outcomes of the base case analysis are shown in Table 2.

**Table 2:**
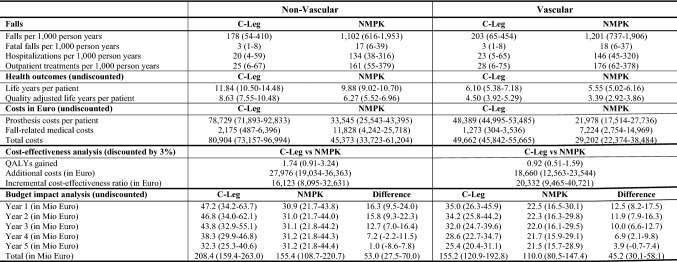
Base case results (including 2.5% and 97.5% quantiles of the probabilistic sensitivity analysis)

*NMPK* non-microprocessor-controlled (mechanical) knee joints, *QALY* quality-adjusted life year

### Cost-effectiveness analysis

**Fig. 2:**
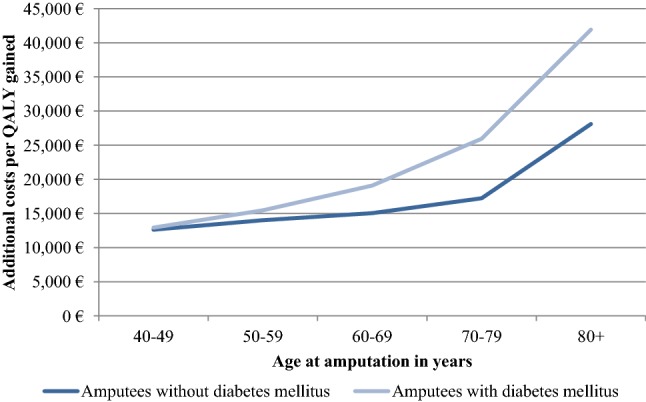
Cost-effectiveness of C-Legs in different age groups

Health outcomes and costs were substantially higher in amputees without DM due to the lower overall survival of patients with DM. Discounted QALY gains of C-Leg usage were 1.74 in patients without DM and 0.92 in patients with DM. Discounted additional costs were 27,976 Euros in amputees without DM and 18,660 Euros in patients with DM. Prosthesis costs were the main cost driver among C-Leg users and constituted around 97% of the overall costs. Among NMPK users, costs of fall-related medical events amounted to 25–26% of total SHI costs (see Table 2). The ICER of C-Legs compared with NMPKs was 16,123 Euros per QALY gained in patients without DM, 20,332 Euros per QALY gained in patients with DM and 17,820 Euros per QALY gained for both patient groups combined. Cost-effectiveness of C-Legs decreased with amputation age. The increase in ICER values intensified in 70+ years old and was more pronounced in patients with DM (see Fig. 2).

### Budget impact analysis

**Fig. 3:**
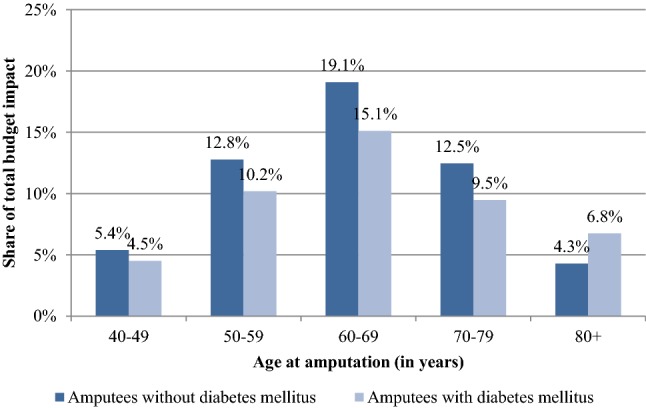
Contribution of different age groups to the budget impact of C-Legs

Results of the budget impact analysis of C-Leg provision showed an increase in SHI costs of around 98 Mio Euro for the period of 2020–2024, 53 Mio Euro in patients without DM and 45 Mio Euro in patients with DM. Around 81% of the additional costs incurred in the first 3 years (2020–2022), when the majority of prosthesis users who received NMPKs before 2020, switch to C-Legs. In addition, increasing costs of NMPK failures—C-Leg failures are covered by a 6-year maintenance package—and higher fall-related medical costs in NMPK users contributed to declining annual budget impacts between 2020 and 2024 (see Table 2). Patients who were between 50 and 79 years old at the time of amputation accounted for around 80% of the total budget impact (see Fig. 3).

**Fig. 4:**
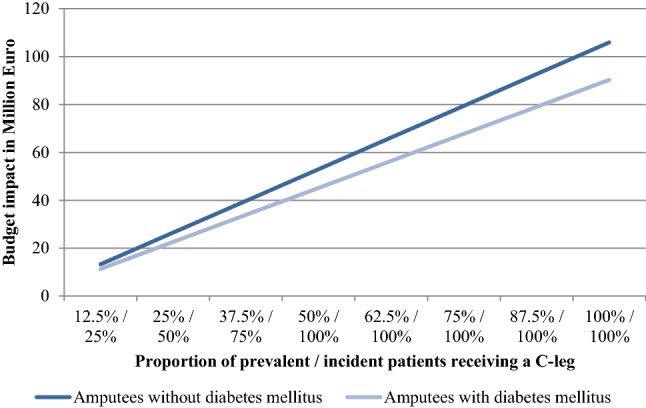
Budget impact of C-Legs for different market shares

Providing C-Legs to all former NMPK users would increase the budget impact to about 196 Mio Euro (106 Mio Euro in prosthesis users without DM and 90 Mio Euro in prosthesis users with DM). If the market share of C-Legs was 25% in all new prosthesis users and only 12.5% of NMPK user switched to C-Legs, additional SHI expenditures would amount to 24.5 Mio Euro (13.2 Mio Euro in prosthesis users without DM and 11.3 Mio Euro in prosthesis users with DM). Budget impacts for different market shares of the C-Leg are presented in Fig. 4.

### Sensitivity analysis

**Fig. 5:**
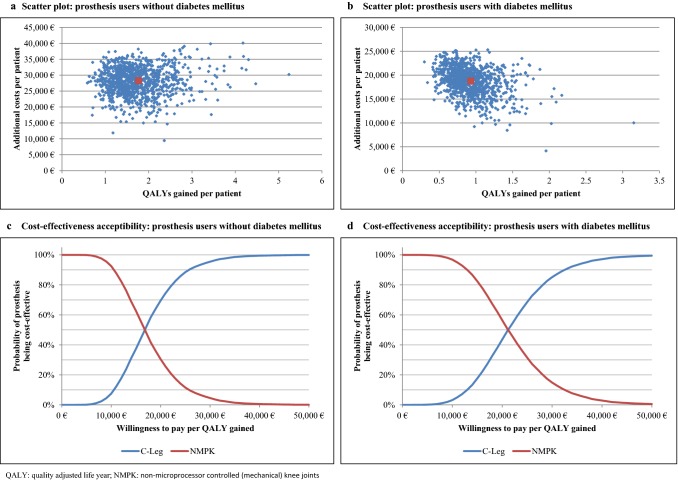
Results of the probabilistic sensitivity analysis

Results of the PSA revealed moderate uncertainty in the ICER estimates and the budget impact of C-Legs (see Fig. 5 and Table 2). The 2.5% and 97.5% quantiles (*Q*_0.025_, *Q*_0.975_) of the ICERs ranged from 8095 Euro to 32,631 Euro per QALY gained in C-Leg user without diabetes and from 9465 Euro to 40,721 Euro per QALY saved in C-Leg user with diabetes. Assuming an ICER threshold of 40,000 Euro (≈ German per CAPITA GDP in 2018) per QALY gained, the probabilities that C-Legs are cost-effective were 99% in amputees without DM and 97% in amputees with DM. At an ICER threshold of 20,000 Euro per QALY gained, probabilities were 70% and 44%, respectively. For both patient groups combined, C-Legs were cost-effective in 59% of the 10,000 PSA iterations at an ICER threshold of 20,000 Euro per QALY gained. The *Q*_0.025_–*Q*_0.975_ intervals of the budget impact of C-Legs ranged from 27.5 to 70.0 Mio Euro in patients without diabetes and from 30.1 to 58.1 Mio Euro for patients with diabetes (Table 2).

**Fig. 6:**
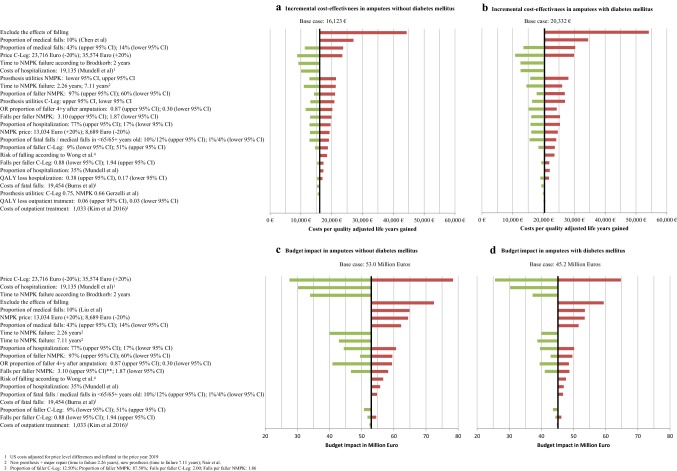
Results of the univariate and multivariate sensitivity analyses

Results of univariate and multivariate sensitivity analyses are shown in Fig. 6. Excluding the effects of falling had the highest impact on the cost-effectiveness of the C-Leg. ICERs for amputees with DM and without DM increased to 54,158 Euro per QALY saved and 44,308 Euro per QALY saved, respectively. The proportion of medical falls and C-Leg prices had also a substantial impact on the cost-effectiveness results. High discount rates reduced the cost-effectiveness of C-Legs. However, the impact of discounting was limited (see supplementary material Table 6).

In the budget impact analyses, changes in cost parameter values (price of C-Legs, hospitalization costs) had the highest impact on the results. Excluding the effects of falling or reducing the proportion of medical falls increased the budget impact of C-Legs substantially. Increasing or decreasing the NMPK survival time resulted in major reductions in the budget impact of C-Legs (see Fig. 6).

## Discussion

This study is the first health economic evaluation of microprocessor-controlled knee prosthesis that analyzed the cost-effectiveness in amputees with different etiologies (DM or no DM). C-Legs provided higher health benefits and had a lower ICER in amputees without DM due to the higher life expectancy of this patient group. However, the higher life expectancy also increased costs and the budget impact of patients without DM. Higher mortality and lower utility values reduced the additional benefits and the cost-effectiveness of C-Legs in older compared with younger amputees. The negative impact of age on the additional benefits and cost-effectiveness of C-Legs has also been reported in an Italian health economic analysis [17]. Overall, the ICER was 16,132 Euro per QALY gained in amputees without DM and 20,332 Euro per QALY gained in amputees with DM.

Several clinical and biomechanical studies [4–15] investigated the safety of the C-Leg compared to NMPKs. Results of these studies reported a reduction in falls and stumbles when patients used C-Legs instead of NMPKs. Our analysis showed that the effects of prosthesis type on falling and fall-related medical events have a major impact on the additional benefits and cost-effectiveness of C-Legs. Excluding these effects would result in a 2.7-fold increase in the ICER estimates according to our study. However, so far, only one other health economic analysis [20] included the medical and financial consequences of falling.

The design of our study is comparable to the analysis of Chen et al. [20] who estimated a base case ICER of 11,606 US-Dollars per QALY gained [20]. Chen et al. applied higher costs of fall-related medical events which was the main reason for the lower ICER compared with our study. Both models predicted comparable incidence rates of fall-related medical events. While we assumed a higher proportion for falls that require medical attention (26.8% derived from Wong et al. [39] vs. 10.4% in Chen et al. [20]), the applied number of falls per faller was lower in our base case analysis due to the exclusion of two outliers from the study sample of Kahle et al. [9]. Chen et al. [10] calculated the proportion of medical falls based on data of the general elderly population. Despite the much lower sample size, we considered results of Wong et al. [25] being more appropriate for our analysis, since the study only included subjects with lower limb amputations. In the sensitivity analysis, we changed the proportion of fall-related medical events to 10.4%. As a result, the ICER increased to 27,100 Euros per QALY gained in amputees without DM and to 34,500 Euros per QALY gained in amputees with DM.

Three other health economic evaluations [16–18] did not include the effect of C-Legs and NMPKs on falling and fall-related events. Brodtkorb et al. reported the lowest base case ICER of all studies and the highest number of QALYs gained. Compared to our analysis and the other studies, Brodtkorb et al. assumed a three- to four-fold higher difference in utilities between the C-Leg and NMPKs [16], which was the main reason for the lower ICER. In the base case analysis, Gerzeli et al. estimated an ICER of 35,971 Euros per QALY gained from the payer’s perspective [18]. The time horizon in the analysis of Gerzeli et al. was 5 years and a 5-year warranty package was assumed for the C-Leg. Hence, C-Leg replacement costs did not incur which may explain the lower ICER compared with our study, when we excluded the effects of falling. We derived utility data from Cutti et al. who reported an ICER of about 51,000 Euros per QALY gained for the age group 65+ years old [17]. The study also had a time horizon of 5 years and effects of falling were not included.

Demonstrating cost-effectiveness is currently not a mandatory requirement for reimbursement in the German SHI. Neither an official cost-effectiveness threshold nor empirical evidence on accepted ICERs do exist for Germany. Therefore, we compared ICER estimates with a threshold proposed by the WHO. WHO-CHOICE considers interventions that avert one DALY (comparable to gains of one QALY) for less than average per capita GDP very cost-effective [54]. At a threshold of 40,000 Euro (≈ average German per Capita GDP in 2018) per QALY gained C-Legs were very likely to be cost-effective. In another very restrictive scenario, we explored the cost-effectiveness of C-Legs at a much lower threshold of 20,000 Euro (≈ half the average German per Capita GDP in 2018) per QALY gained. In this case, the probability that C-Legs were cost-effective decreased to 70% in amputees without DM and to 44% in amputees with DM. For both patient groups combined, the probability that C-Legs are cost-effective at a threshold of 20,000 Euro per QALY gained was 59%.

The main limitations of our study are the lack of German utility values for prosthesis users, long-term data on the risk of falling and health economic consequences of fall-related events as well as the parameter uncertainty regarding the risk and the medical consequences of falling in prosthesis users. Comparative studies that have investigated the risk of falling in prosthesis users [7, 9–12] had rather short follow-ups and small sample sizes. The only study that investigated fall consequences in prosthesis users [39] also had a small sample size. Small sample sizes typically result in high parameter uncertainties and the lack of long-term data induces additional uncertainty. Hence, future research should focus on conducting comparative trials with larger sample sizes and longer trial durations. Establishing a clinical leg prosthesis register in Germany could also be very useful for the collection of more robust input data for health economic evaluations. Utility estimates were relatively stable across studies [16–18]. We used utility data from the most recent study [17] with the lowest utility difference between the C-Leg and NMPKs.

We did not include economic- and health-related long-term consequences of medical fall events in our analysis. The inclusion of long-term costs and long-term disutility of severe fall injuries would increase the benefits of the C-Leg and would improve its cost-effectiveness due to the superior safety of microprocessor-controlled knees compared with NMPKs. Adopting the German societal instead of the SHI perspective is likely to further improve the health economic outcomes of the C-Leg compared with NMPKs. Gerzeli et al. [18] reported a substantial reduction of indirect costs in C-Leg user compared with NMPK user. As a result, the C-Leg dominated the NMPK from the societal perspective, while the ICER from the payer’s perspective was around 36,000 Euros per QALY gained.

Considering the results of the base case and sensitivity analyses of our study, the C-Leg is likely to be cost-effective compared to NMPKs in Germany, when applying cost-effectiveness thresholds proposed by WHO-CHOICE. However, larger comparative trials and comprehensive German health economic data are required to provide more robust evidence regarding the cost-effectiveness of C-Legs.

## Conclusion

The results of our study suggest that the C-Leg provides substantial additional benefits and is very likely to be cost-effective in transfemoral amputees with and without DM from the perspective of the German SHI, when adopting an ICER threshold of around 40,000 Euro per QALY gained. For patients without DM and for both patient groups combined, C-Legs may also be cost-effective at a threshold of 20,000 Euro per QALY saved.

## Electronic supplementary material

Below is the link to the electronic supplementary material.

Supplementary material 1 (DOCX 837 kb)
