# Supplementary material for: Cost-effectiveness and budget impact of the microprocessor-controlled knee C-Leg in transfemoral amputees with and without diabetes mellitus
Source: Eur J Health Econ. 2020 Jan 2;21(3):437–49. doi: 10.1007/s10198-019-01138-y (PMC7188726; doi:10.1007/s10198-019-01138-y)
Supplement: Supplementary file 1 — Supplementary material 1 (DOCX 837 kb) [file 10198_2019_1138_MOESM1_ESM.docx]

**Supplementary Material**

[1 Annual incidence of transfemoral amputations and estimated number of leg prosthesis users 2](#_Toc24404596)

[2 Estimation of fall costs 9](#_Toc24404597)

[3 Impact of discount rates on the cost-effectiveness of the C-Leg 11](#_Toc24404598)

[4 References 12](#_Toc24404599)

# Annual incidence of transfemoral amputations and estimated number of leg prosthesis users

In order to calculate the budget impact for the period 2020-2024, the number of annual incident leg prosthesis users had to be determined. Surviving incident leg prosthesis users of the period 2000-2019 constituted the prevalent patient cohorts in the budget impact model.

We performed a linear regression on the logged incidence rates of transfemoral amputations in Germany in 2005-2017. Data was obtained from official German DRG statistics (OPS codes 5-864.3-5-864.7). [1] Time trends in the incidence for the period 2005-2011 was used to predict the incidence for 2000-2005 and time trends in the incidence for the period 2012-2017 was used to project incidences for 2018-2024. Time trends are shown in tables 1 and 2, including parameters of the distribution applied in the probabilistic sensitivity analysis. A time trend of 0.955 means that the incidence decreases by 4.45% each following year.

Case numbers were calculated using demographic data. [2, 3] Finally, the annual number of incident prosthetic users was determined as follows:

$${IPU}_{noDM}=TA\cdot\left( {1-pTA}_{DM} \right)\cdot\left( 1-{mTA}_{noDM} \right)\cdot pSF\cdot pSHI$$

$${IPU}_{DM}=TA\cdot{pTA}_{DM}\cdot\left( 1-{mTA}_{DM} \right)\cdot pSF\cdot pSHI$$

IPU_noDM_: incident prosthesis users without DM

IPU_DM_: incident prosthesis users with DM

TA: transfemoral amputations

pTA_DM_: proportion of transfemoral amputees with DM

mTA_noDM_: 30 day mortality in amputees without DM

mTA_DM_: 30 day mortality in amputees with DM

pSF: proportion of successful fitting

pSHI: proportion of Germans in the SHI

Observed and projected annual incidences of transfemoral amputations in Germany are presented in figure 1. Estimated annual numbers of incident prosthesis users without diabetes mellitus (DM) are shown in figure 2 and users with DM in figure 3. Figure 4 and 5 present the total annual number of leg prosthesis user (incident + prevalent user) of amputees without diabetes and amputees with diabetes respectively.

Table 1: Time trends in the incidence of transfemoral amputations 2005-2011

| **Age group** | **Estimate** | **95% CI** | **Distribution** | | |
| --- | --- | --- | --- | --- | --- |
|  |  |  | **Type** | **Parameter** | |
| 40-49 years old | 0.955 | 0.931-0.979 | lognormal | μ=-0.046 | σ=0.013 |
| 50-59 years old | 0.959 | 0.946-0.973 | lognormal | μ=-0.042 | σ=0.009 |
| 60-69 years old | 0.955 | 0.943-0.966 | lognormal | μ=-0.046 | σ=0.006 |
| 70-79 years old | 0.932 | 0.923-0.942 | lognormal | μ=-0.070 | σ=0.005 |
| 80+ years old | 0.935 | 0.925-0.944 | lognormal | μ=-0.067 | σ=0.005 |

Table 2: Time trends in the incidence of transfemoral amputations 2012-2017

| **Age group** | **Estimate** | **95% CI** | **Distribution** | | |
| --- | --- | --- | --- | --- | --- |
|  |  |  | **Type** | **Parameter** | |
| 40-49 years old | 1.030 | 1.017-1.044 | lognormal | μ=0.030 | σ=0.007 |
| 50-59 years old | 0.984 | 0.962-1.006 | lognormal | μ=-0.016 | σ=0.011 |
| 60-69 years old | 0.999 | 0.983-1.015 | lognormal | μ=-0.001 | σ=0.008 |
| 70-79 years old | 0.960 | 0.937-0.984 | lognormal | μ=-0.041 | σ=0.013 |
| 80+ years old | 0.923 | 0.911-0.935 | lognormal | μ=-0.080 | σ=0.007 |

Figure 1: Observed and projected incidences of transfemoral amputations in Germany


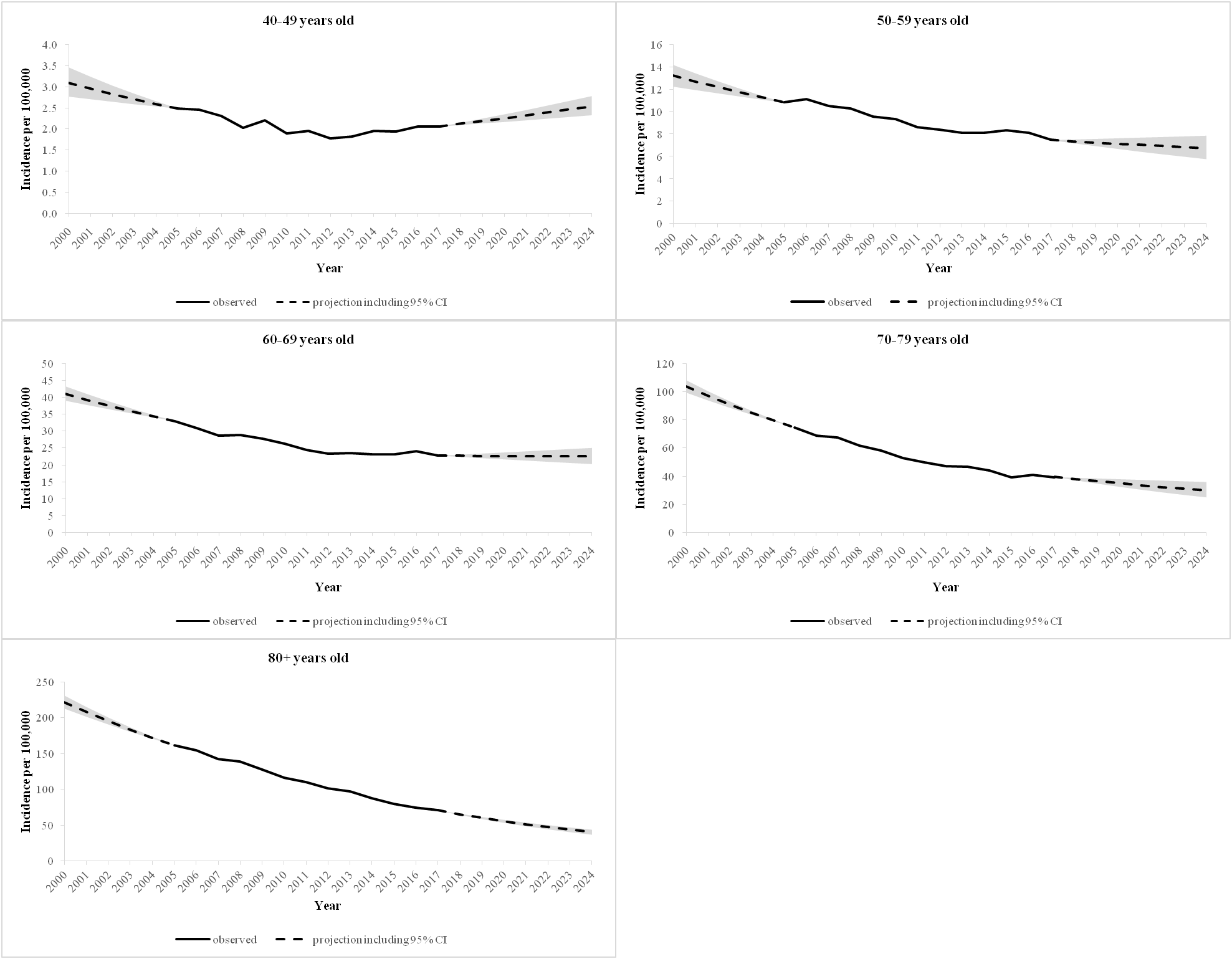


Figure 2: Estimated annual incident leg prosthesis users without diabetes mellitus (mean and 95% CI)


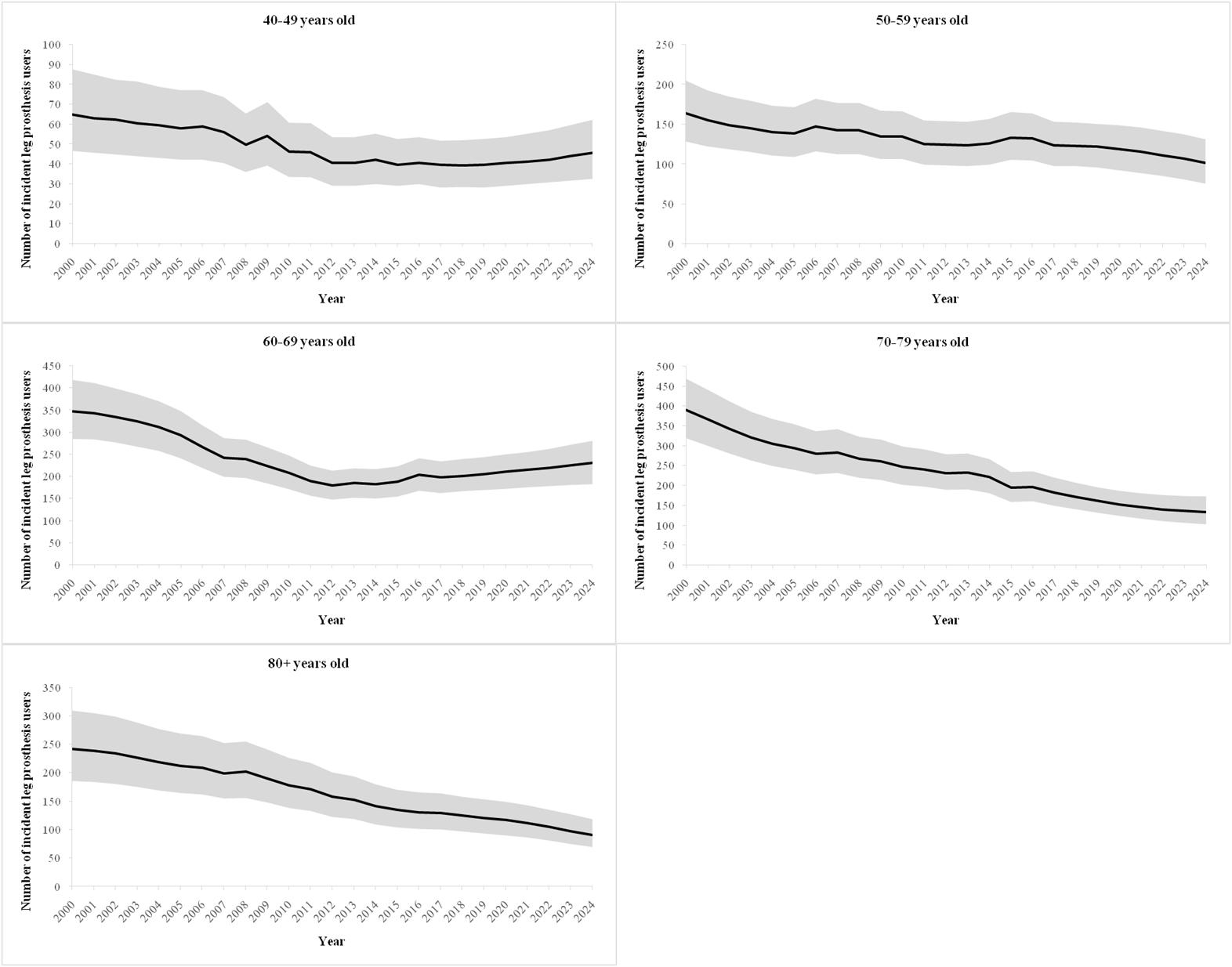


Figure 3: Estimated annual incident leg prosthesis users with diabetes mellitus (mean and 95% CI)


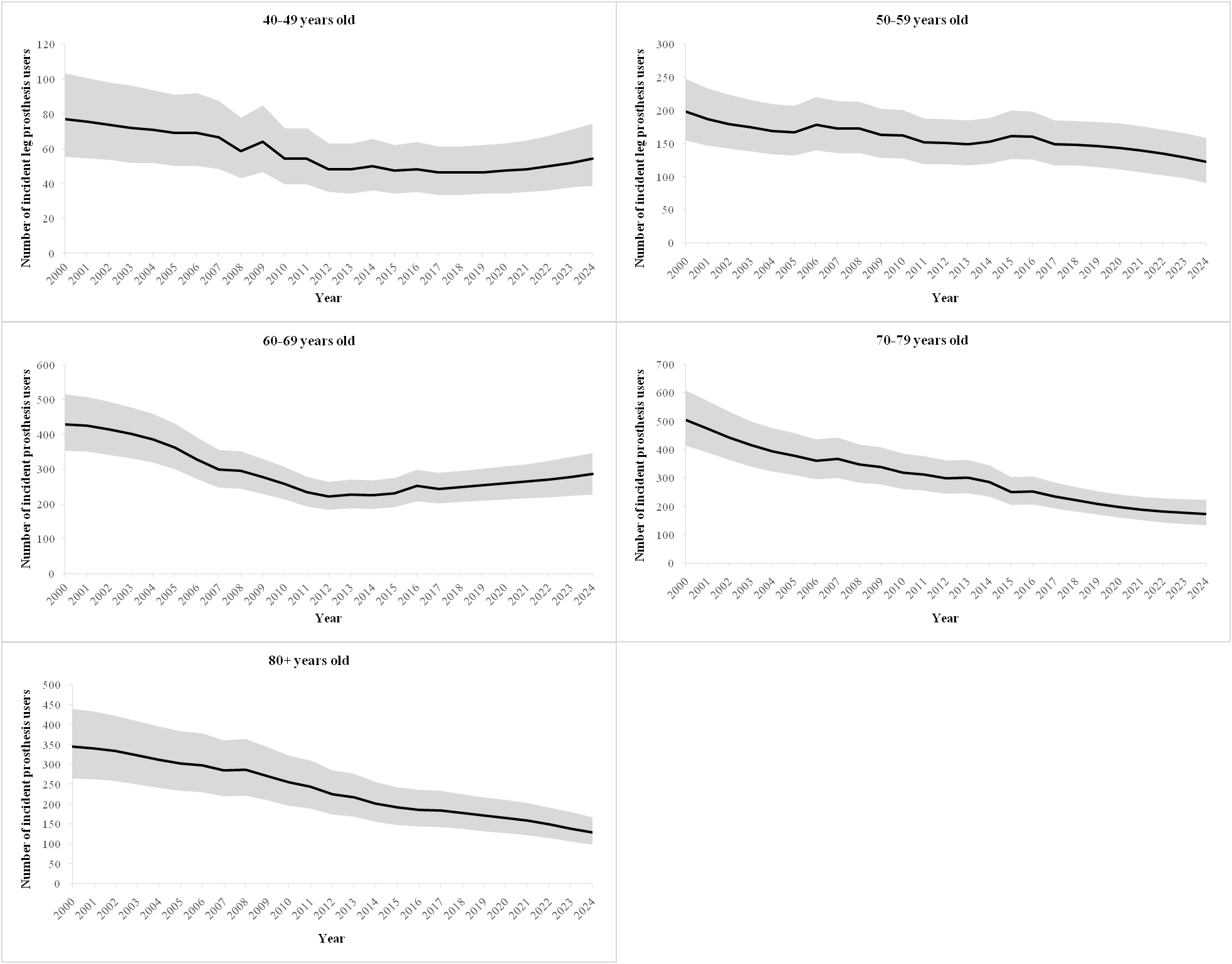


Figure 4: Estimated (incident + prevalent) leg prosthesis users without diabetes mellitus (mean and 95% CI)


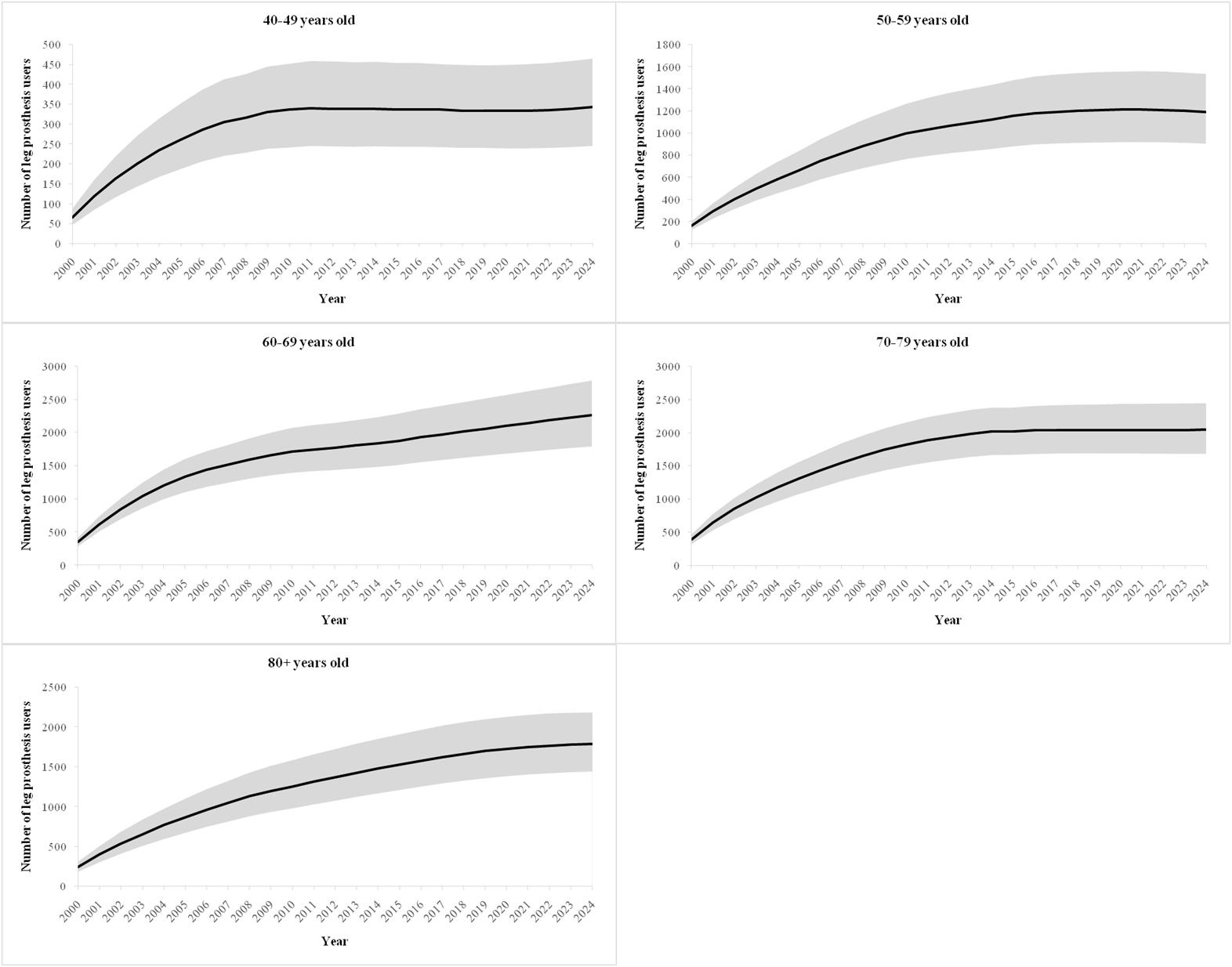


Figure 5: Estimated (incident + prevalent) leg prosthesis users without diabetes mellitus (mean and 95% CI)


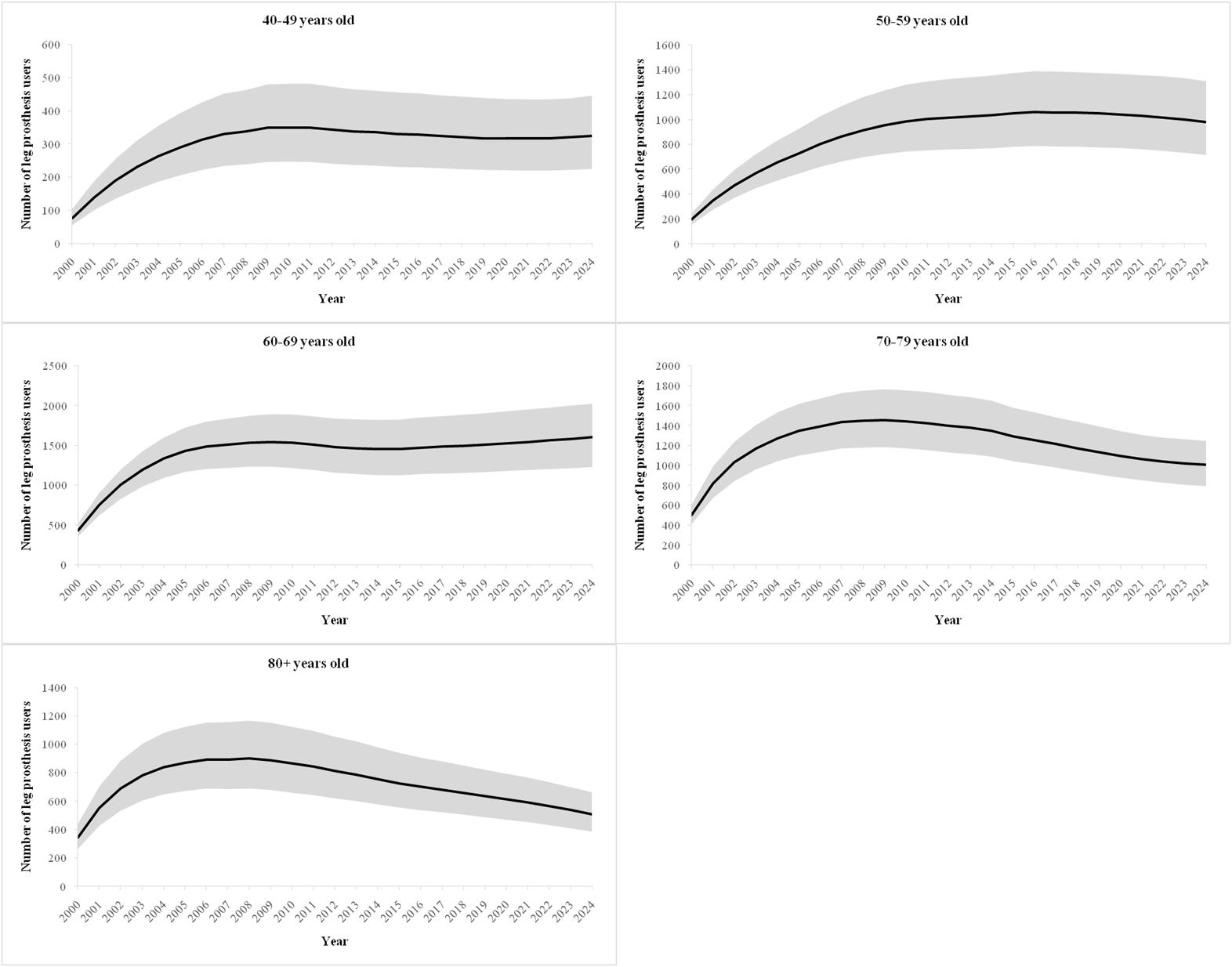


# Estimation of fall costs

Fall injury types were defined based on a study by Hartholt et al. [4] Costs of hospitalized patients included the costs of hospital treatments, inpatient rehabilitation treatment and outpatient treatment of hospitalization. [5] Costs of hospital treatments per case (table 3) were calculated based on German DRGs (G-DRG). [6] Mean G-DRG costs per case (table 3) were the weighted mean of all DRGs that were reported for the ICD10 codes of a defined fall injury [6]. The distribution of fall injuries in hospitalized patients (table 3) was obtained from Hartholt et al. [4]

Costs for inpatient rehabilitation treatment (table 4) were calculated by multiplying the mean costs of inpatient rehabilitation per day (127.82 Euro; costs of 2016 [7] adjusted for inflation [7, 8]) by the mean length of stay [9]. The rehabilitation probability after hospitalization (table 4) was calculated by dividing the number of hospitalized cases in 2017 [10] by the number of inpatient rehabilitation cases in 2017 [9].

Outpatient treatment costs after hospitalization and costs for exclusive outpatient treatment were obtained from a health economic study on the costs of osteoporosis in Germany [5] and adjusted for inflation [8]. The distribution of fall injuries in exclusive outpatient treatments were taken from Hartholt et al. [4]

Since German cost data of fatal falls did not exist, we assumed that they were equal to the costs for hospital treatments. This was a rather conservative assumption considering that US health economic studies reported that costs of fatal falls were comparable to costs of hospitalized patients. [11, 12]

Table 3: Costs for hospital treatments of fall injuries

| **Injury** | **ICD10** | **% of patients[4]** | **Mean G-DRG cost per case[6]** | **Distribution** | | |
| --- | --- | --- | --- | --- | --- | --- |
|  |  |  |  | **Type** | **Parameter** | |
| Superficial injuries | S40, S50, S60, S70, S80, S90 | 7.7% | 2,250 € | lognormal | μ=7.719 | σ=0.011 |
| Hip fracture | S72.0-S72.2 | 52.2% | 7,476 € | lognormal | μ=8.919 | σ=0.003 |
| Wrist/hand fracture | S62 | 2.6% | 2,801 € | lognormal | μ=7.938 | σ=0.007 |
| Shoulder/upper arm fracture | S42 | 4.5% | 4,897 € | lognormal | μ=8.496 | σ=0.004 |
| Lower leg/ankle fracture | S82 | 6.3% | 4,823 € | lognormal | μ=8.481 | σ=0.005 |
| Wound head/face | S00-S01 | 3.9% | 1,451 € | lognormal | μ=7.280 | σ=0.010 |
| Skull/brain inury | S02 | 8.3% | 3,355 € | lognormal | μ=8.118 | σ=0.012 |
| Elbow/forearm fracture | S52 | 2.0% | 3,345 € | lognormal | μ=8.115 | σ=0.004 |
| Other femur fracture | S72.3-S72.9 | 8.0% | 7,558 € | lognormal | μ=8.930 | σ=0.009 |
| Foot/toe fracture | S92 | 0.3% | 3,333 € | lognormal | μ=8.112 | σ=0.011 |
| Pelvis fracture | S32.1-S32.5 | 4.2% | 5,044 € | lognormal | μ=8.526 | σ=0.012 |

Table 4: Costs for inpatient rehabilitation treatment of fall injuries

| **Injury** | **ICD10** | **Rehabilitation cost per case[7, 9]** | **Length of stay (in days)[9]** | **Reha probability after hospitalization^3^** |
| --- | --- | --- | --- | --- |
| Superficial injuries | S40, S50, S60, S70, S80, S90 | 3,060 € | 23.94 | 0.50% |
| Hip fracture | S72.0-S72.2 | 2,773 € | 21.70 | 18.10% |
| Wrist/hand fracture | S62 | 3,143 € | 24.59 | 0.80% |
| Shoulder/upper arm fracture | S42 | 2,820 € | 22.06 | 7.00% |
| Lower leg/ankle fracture | S82 | 3,213 € | 25.13 | 4.90% |
| Wound head/face | S00-S01 | 2,890 € | 22.61 | 0.10% |
| Skull/brain inury | S02 | 3,382 € | 26.46 | 0.90% |
| Elbow/forearm fracture | S52 | 3,105 € | 24.29 | 0.70% |
| Other femur fracture | S72.3-S72.9 | 3,009 € | 23.54 | 21.30% |
| Foot/toe fracture | S92 | 3,329 € | 26.04 | 2.90% |
| Pelvis fracture | S32.1-S32.5 | 2,909 € | 22.76 | 6.10% |

Table 5: Costs for outpatient treatment of fall injuries

| **Injury** | **ICD10** | **Outpatient treatment cost after hospitalization / case[5]** | **Exclusive outpatient treatment** | |
| --- | --- | --- | --- | --- |
|  |  |  | **Cost per case[5]** | **% of patients[4]** |
| Superficial injuries | S40, S50, S60, S70, S80, S90 | 442.17 €^1^ | 471.12 €^1^ | 33.8% |
| Hip fracture | S72.0-S72.2 | 1,087.40 € | 1,087.40 € | 1.6% |
| Wrist/hand fracture | S62 | 884.35 € | 942.25 € | 25.9% |
| Shoulder/upper arm fracture | S42 | 884.35 € | 942.25 € | 13.0% |
| Lower leg/ankle fracture | S82 | 884.35 € | 942.25 € | 5.8% |
| Wound head/face | S00-S01 | 884.35 €^2^ | 942.25 €^2^ | 6.8% |
| Skull/brain inury | S02 | 884.35 €^2^ | 942.25 €^2^ | 2.5% |
| Elbow/forearm fracture | S52 | 884.35 € | 942.25 € | 4.6% |
| Other femur fracture | S72.3-S72.9 | 1,087.40 € | 1,087.40 € | 0.3% |
| Foot/toe fracture | S92 | 884.35 € | 942.25 € | 3.9% |
| Pelvis fracture | S32.1-S32.5 | 1,087.40 € | 1,087.40 € | 1.8% |
| ^1^ Assumption: costs of superficial injuries were 50% arm or lower leg fractures; ^2^ Assumption: costs of wounds of the head/face or skull/brain injuries were comparable to the costs of arm or lower leg fractures. | | | | |

# Impact of discount rates on the cost-effectiveness of the C-Leg

Table 6: Impact of discount rates on the incremental cost-effectiveness ratio

| **Discount rates** | **Prosthesis users without DM** | **Prosthesis users with DM** |
| --- | --- | --- |
| **Base case: QALY 3%; costs 3%** | **16,123 Euro per QALY gained** | **20,332 Euro per QALY gained** |
| QALY 0%; costs 0% | 15,187 Euro per QALY gained | 18,573 Eurp per QALY gained |
| QALY 1.5%; costs 1.5% | 15,605 Euro per QALY gained | 19,435 Euro per QALY gained |
| QALY 1.5%; costs 3% | 16,948 Euro per QALY gained | 21,565 Euro per QALY gained |
| QALY 5%; costs 5% | 13,978 Euro per QALY gained | 18,641 Euro per QALY gained |
| DM: diabetes mellitus; QALY: quality adjusted life years gained | | |

References

1. Federal Statistical Office of Germany: DRG-Statistik1 2005 - 2017. Vollstationäre Patientinnen und Patienten in Krankenhäusern (einschl. Sterbe- und Stundenfälle), ausgewählte Operationen2) 5-864ff auf Ebene des OPS Endstellers. http://www.gbe-bund.de (2019). Accessed 25 September 2019

2. Federal Statistical Office of Germany: Bevölkerung: Deutschland, Stichtag, Altersjahre. https://www-genesis.destatis.de/genesis/online (2019). Accessed 25 September 2019

3. Federal Statistical Office of Germany: Vorausberechneter Bevölkerungsstand: Deutschland, Stichtag, Varianten der Bevölkerungsvorausberechnung, Geschlecht, Altersjahre. https://www-genesis.destatis.de/genesis/online (2019). Accessed 25 September 2019

4. Hartholt, K.A., van Beeck, E.F., Polinder, S., van der Velde, N., van Lieshout, E.M.M., Panneman, M.J.M., van der Cammen, T.J.M., Patka, P.: Societal consequences of falls in the older population. Injuries, healthcare costs, and long-term reduced quality of life. The Journal of trauma (2011). https://doi.org/10.1097/TA.0b013e3181f6f5e5

5. Bleibler, F., Rapp, K., Jaensch, A., Becker, C., König, H.-H.: Expected lifetime numbers and costs of fractures in postmenopausal women with and without osteoporosis in Germany: a discrete event simulation model. BMC health services research (2014). https://doi.org/10.1186/1472-6963-14-284

6. Institut für das Entgeldsystem im Krankenhaus (InEK): G-DRG-Reportbrowser 2019. https://www.g-drg.de. Accessed 29 September 2019

7. Deutsche Rentenversicherung: Reha-Bericht 2018. https://www.reha-berichte-drv.de (2018). Accessed 29 September 2019

8. Federal Statistical Office of Germany: Verbraucherpreisindizesfür Deutschland. https://www-genesis.destatis.de/genesis/online (2019). Accessed 25 September 2019

9. Federal Statistical Office of Germany: Diagnosedaten der Patientinnen und Patienten in Vorsorge- oder Rehabilitationseinrichtungen. www.gbe-bund.de (2019). Accessed 25 September 2019

10. Federal Statistical Office of Germany: Diagnosedaten der Krankenhäuser. www.gbe-bund.de (2019). Accessed 25 September 2019

11. Burns, E.R., Stevens, J.A., Lee, R.: The direct costs of fatal and non-fatal falls among older adults - United States. Journal of safety research (2016). https://doi.org/10.1016/j.jsr.2016.05.001

12. Chen, C., Hanson, M., Chaturvedi, R., Mattke, S., Hillestad, R., Liu, H.H.: Economic benefits of microprocessor controlled prosthetic knees. A modeling study. Journal of neuroengineering and rehabilitation (2018). https://doi.org/10.1186/s12984-018-0405-8
